# Therapeutic Strategies and Potential Actions of Female Sex Steroid Hormones and Their Receptors in Colon Cancer Based on Preclinical Studies

**DOI:** 10.3390/life12040605

**Published:** 2022-04-18

**Authors:** Amani A. Mahbub

**Affiliations:** Laboratory Medicine Department, Faculty of Applied Medical Sciences, Umm Al-Qura University, P.O. Box 715, Makkah 21955, Saudi Arabia; aamahbob@uqu.edu.sa

**Keywords:** colon cancer, female sex steroid hormones, estrogen, estradiol, progesterone, estrogen receptor, progesterone receptor

## Abstract

Several epidemiological studies have reported that the use of female sex steroid hormones could reduce the risk of colon cancer (CRC). This review summarizes the available data related to estradiol (E2) and progesterone (P4) single and dual treatments in CRC male and female in vitro and in vivo models, mainly from preclinical studies, alongside their potential molecular mechanisms. Most of the studies showed that E2 exogenous treatment and/or reactivation of its beta receptor (ERβ) significantly inhibited cell proliferation, induced cell cycle arrest, and promoted apoptosis by modulating several molecular pathways. Likewise, the inhibition of ERα receptors produced similar antitumorigenic actions, both in vivo and in vitro, suggesting that E2 could have dual opposing roles in CRC that are dependent on the expression profile of its nuclear receptors. The available studies on P4 are scarce, and the results revealed that in vitro and in vivo treatments with natural and synthetic progesterone were also associated with promising tumoricidal actions. Nevertheless, the combination of E2 with P4 showed enhanced anticancer activities compared with their monotherapy protocols in male–female cell lines and animals. Collectively, the studies suggested that the female sex steroid hormones could provide a novel and effective therapeutic strategy against CRC.

## 1. Introduction

Colon cancer (CRC) is a heterogeneous disease with different phenotypes distinguished by their specific clinical and molecular characteristics [1,2,3,4,5]. The development of CRC results from the stepwise accumulation of a sequence of genetic and epigenetic alterations in normal colonic epithelium, leading to adenomas and invasive adenocarcinomas [5]. Globally, CRC oncogenesis includes three genetic and epigenetic abnormalities: (1) chromosomal instability (CIN); (2) methylation of CpG island methylator phenotype (CIMP); and (3) instability of microsatellite DNA regions (MSI) [5]. The most common gene mutations seen in CRC are adenomatous polyposis coli (APC), catenin beta 1 (CTNNB1), Kirsten Ras (KRAS), B-Raf (BRAF), small mothers against decapentaplegic homolog 4 (SMAD4), transforming growth factor beta receptor 2 (TGFBR2), tumor protein 53 (TP53), and phosphatidylinositol-4,5- bisphosphate 3-kinase catalytic subunit-alpha (PIK3CA) [6,7,8]. These genetic alterations promote colon carcinogenesis by disturbing the function of key signaling pathways—including the Wingless/Int (Wnt)–β-catenin, epidermal growth factor receptor (EGFR), vascular endothelial growth factor (VEGF), RAS–RAF–mitogen-activated protein kinase (MAPK), transforming growth factor-beta (TGF-β), phosphatidylinositol-4,5-bisphosphate 3-kinase (PI3K)–alpha serine/threonine protein kinase (AKT) signaling pathways—that stimulate cell cycle progression, cell proliferation, and metastasis, whilst inhibiting DNA repair and apoptosis [1,5,6,7,8,9,10,11,12,13]. Hence, targeting the genetic alterations and their dysregulated molecular pathways is essential for developing more efficient therapeutic targets for CRC treatment.

Although considerable improvements in the therapeutic strategies against CRC have been made, the rates of metastasis, as well as relapse, are increasing due to presentation at late stages of malignancy, development of chemoresistance, and occurrence of severe side effects during treatment [14,15]. Chemoresistance is the primary reason for treatment failure and occurs in more than 90% of CRC patients with metastatic tumors [14]. Current researchers are still searching for alternative therapies for CRC to overcome these clinical problems and/or to increase survival rates and quality of life. Till now, CRC remains one of the leading causes of cancer-associated morbidity and mortality in males and females worldwide [16]. Nevertheless, the incidence rate of CRC is lower in premenopausal females and linked with better 5-year survival rates compared with age-matched males [5,17,18,19,20,21,22,23].

Indeed, oophorectomy and the early suppression of female sex hormones have been revealed to increase the risk of CRC by 30% [24]. Other studies also reported that premenopausal CRC patients had higher 5-year survival rates than males of the same age or postmenopausal women; further proposing that female sex steroid hormones could improve prognosis [22,25]. In this regard, age is considered to be a major risk factor for CRC, with an estimated 90% of cases and 94% of deaths occurring in individuals older than 50 years [19,22,23]. Moreover, a meta-analysis of 13 retrospective cohort studies and 1 randomized controlled trial in 2017 revealed that survival in CRC patients was gender-dependent [26]. Another recent German population-based cohort study that included 185,967 patients also demonstrated significantly better overall and recurrence-free survival in females than males [27]. Hence, the differences related to the patient’s gender and age of CRC onset could be associated with abnormal alterations in systemic and/or colonic levels of ovarian sex steroids (i.e., estrogens and progesterone) [18,19,20,21,22,23,28,29].

### 1.1. Female Sex Steroid Hormones

Sex steroid hormones are derived from cholesterol and involve estrogens (e.g., 17β-estradiol, estriol, and estrone), progestogens (e.g., progesterone), and androgens (e.g., testosterone) [22]. While sex hormones are mainly produced by female ovarian and male testicular tissues, there are other tissues, including adipose tissue, adrenal glands, skin, brain, and bone, which also contribute to the production of sex hormones [22,30,31]. In plasma, sex hormones are primarily bound to albumin and sex-hormone-binding globulin (SHBG), whilst only 1–3% of those hormones are free and biologically active [22]. Both genders produce all types of sex hormones but in differential amounts, and their biological effects depend on their systemic concentrations, expression of their nuclear receptors, and their specific interactions with target organs [22]. Commonly, estrogens and progesterone serum concentrations are higher in females than males and play several roles in female physiology, including the menstrual cycle and pregnancy in premenopausal females, whereas the production of both hormones from the ovaries declines to extremely low levels in postmenopausal females; postmenopausal levels correspond to male estrogen and progesterone levels [32,33,34]. Based on the current epidemiological data, estrogens and/or progesterone could play a role in preventing CRC (Section 1.2). 

Progestogens are the first products of cholesterol side cleavage in gonadal tissues during sex hormones synthesis [35,36,37], and the most potent family member is progesterone (P4) [35,36,37]. On the other hand, there are three natural estrogens, known as estrone (E1), 17β-estradiol (E2), and estriol (E3) [22]. The most potent member of the estrogen family which is abundant in humans is E2 [19,22,23], which is chiefly generated by reproductive tissues, such as ovarian follicles, placenta, and corpus luteum [23]. The actions of E2 and progesterone are dependent on binding to their specific nuclear receptors (ERs and PRs, respectively) [33,38], which act as transcription factors to stimulate genomic and non-genomic actions that often converge at the adjacent responsive genes [33,38,39].

ERs are classified into alpha (ERα) and beta (ERβ) subtypes, and both contribute to the initiation, development, and progression of hormone-dependent neoplasms [38]. Moreover, E2 could produce opposing actions in cancer biology that are dependent on the type of activated ER [19,23]. In further detail, E2 induces ERα-dependent cell cycle progression, cell proliferation, inflammation, and carcinogenesis [19,23,38,40]. In contrast, the activation of ERβ by E2 provokes cell cycle arrest, modulates the expression of many of the ERα-regulated genes, and induces multiple anticancer activities [19,23,38,40]. Similarly, PR has two isoforms—the N-terminal-truncated A (PRA) and the full-length B (PRB) [33]—and the activation of both isoforms by P4 causes a structural change following segregation and dimerization of heat shock proteins, which bind to specific DNA promoter sites as P4 response elements, causing transcriptional activation or repression [33]. 

### 1.2. E2 and P4 in CRC: Epidemiological Findings

Numerous epidemiological [29,41,42] and randomized controlled trials (RCTs) [29,43,44,45,46,47,48] studies have found that exogenous treatment with oral contraceptives (OC) and hormone replacement therapy (HRT) were linked with protective roles against CRC development. Under this theme, women using OC had a 10–20% lower risk of developing CRC [41]. Concurrently, others have demonstrated that menopausal females using HRT had significantly lower rates of CRC, by almost 50–63%, compared with age-matched females who were not using HRT; these results imply that female sex steroids hormones could protect against CRC [49,50,51,52,53,54,55]. Likewise, the use of HRT for a few years prior to CRC diagnosis in females was associated with improved survival rates [56,57,58]. Furthermore, several meta-analysis studies revealed that HRT reduced the risk of developing CRC in microsatellite-stable states [42], as well as improving mortality rates in female CRC patients [59,60]. In addition, Labadie et al. (2020) [61] has also confirmed the strong inverse correlation between HRT use and overall CRC risk in postmenopausal females, suggesting efficient chemopreventive actions for HRT by inhibiting adenoma–carcinoma in CRC. In contrast, a few epidemiological studies have failed to detect associations between the HRT and CRC incidence in postmenopausal females [62,63,64,65].

The effects of combined estrogen and progesterone therapy and CRC development in postmenopausal females was also studied, and data from the Women’s Health Initiative revealed that dual hormonal therapy in postmenopausal females was associated with a reduction in CRC incidence by 38% [53]. Similarly, Chlebowski et al. (2004) [28] has demonstrated that combining conjugated equine estrogens at 0.625 mg/day and medroxyprogesterone acetate (MPA) at 2.5 mg/day decreased the incidence of CRC by 40% in postmenopausal women aged between 50 and 79 years. In contrast, treatment with conjugated equine estrogens alone showed no effects on CRC incidence [28], suggesting that estrogen monotherapy has limited anticancer activity, whereas the addition of progesterone could have more potent chemopreventive actions against CRC [28,66]. Although Labadie et al. (2020) [61] reported that estrogen, both alone and in combination with progestin, reduced the risk of CRC, they proposed that the protective roles were mediated by E2, not progestin, in CRC. 

Hence, a better understanding of the roles of female sex steroid hormones and their related receptors in CRC could be a promising approach for the prevention, early diagnosis, and/or treatment development of CRC, thus improving treatment and survival outcomes, as well as the quality of life in patients diagnosed with CRC. 

### 1.3. Review Aim

This review, therefore, aimed to summarize the currently available preclinical experiments that focused on the potential beneficial effects of female sex steroid hormones (i.e., estrogen and progesterone) and their related receptors in the treatment of colon cancer cell lines/animal models; additionally, this review aimed to determine the molecular mechanisms underlying their tumoricidal actions against colon neoplasia in both genders. 

### 1.4. Method

A literature search was carried out in PubMed for obtaining information related to the therapeutic strategies and potential actions of female sex steroid hormones (estrogen and progesterone, alone and in combination) and their related receptors in colon cancer based on preclinical studies. The keywords for the literature search were the following: colon/colorectal cancer, female sex steroid hormones, therapeutic potential effect/molecular mechanisms of estrogen, estradiol, estrogen receptor, progesterone, progesterone receptor, endogenous expression of estrogen receptor, endogenous expression of progesterone receptor, in vitro human male and female CRC cell lines, and in vivo CRC animal models. 

## 2. Endogenous Expression of Female Sex Steroid Hormone Receptors in Normal and Malignant Colon

### 2.1. Endogenous Expression of ERα/β in Colonic Tissues and Cells

Earlier studies demonstrated that ER has endogenously low expression in normal colonic and malignant colonic tissues, but without determining the receptor isoforms [67,68,69,70]. Consequent studies then noted that ERβ is the most abundantly expressed receptor in normal colonic tissues [71,72,73]. However, both ERα and β were detected in CRC clinical samples, animal models, and in various colon cancer cell lines, with controversial results regarding their roles in colon carcinogenesis. In detail, in vivo studies revealed that deletion of any of the ER subtypes in mice promoted CRC development and progression, suggesting that both receptors are essential for maintaining the integrity of colonic tissue [74]. In contrast, the ERα subtype is highly expressed in clinical malignant colonic specimens, proposing that the receptor expression status could be a potentially sensitive and specific prognostic marker [75]. Cavallini et al. (2005) [76] has also found that estrogen-receptor-related receptor-α (ERRα) was highly expressed in tumorous tissues compared with their corresponding adjacent normal colon tissues. Others also demonstrated that ERα produced different activities during colon carcinogenesis, which could be dependent on the existence of several receptor splice forms, among which the ERα46 induced antiproliferative and apoptotic actions, whilst the shorter variant, ERα36, was promoted CRC progression [77]. The authors have also suggested that ERα46 receptor isoform could represent a potential target for CRC treatment [77].

Remarkably, cancerous colon tissues had reduced expression of ERβ subtype compared with normal colon mucosa [71,78,79,80,81]; additionally, the receptor status correlated negatively with poor differentiation [79], advanced Dukes’ staging [18], and depth of invasion [82]. Therefore, these studies suggested that estrogens may induce antitumorigenic effects through β, not α, receptors in colonic tissue [71,72,80,83,84,85,86]. In agreement, Topi et al. also found that high ERβ expression in female CRC patients associated independently with improved prognosis [87] and lower metastases [88] compared with patients with low ERβ expression. Thus, ERβ seems to have chemopreventive actions against CRC development and progression, which may provide a ground for developing alternative diagnostic tools and novel approaches for the prevention and/or treatment of CRC.

Additionally, ERβ was naturally expressed in nine immortalized human male (Table 1) and three female (Table 2) colon cancer cell lines, whereas most of these cell lines lacked the expression of ERα. Moreover, other ERβ isoforms have also been demonstrated in colon cancer cell lines (Table 1 and Table 2).

### 2.2. Endogenous Expression of PRs (A/B) in Colonic Tissues and Cells

There are contradictory findings regarding the roles of both PR receptor (A/B) in normal and malignant colonic tissues/cells, which could be related to using different techniques/methods for detecting the receptors in normal and malignant colonic specimens, as well as differences in study populations and/or discrepancies in CRC cell lines [67,68,69,70,71,72,73,74,75,76,77,78,95,96,97,98,99]. In this regard, earlier studies reported low expression of PR in normal and malignant colonic tissues [67,68,69,70], as well as in human female (HT29, COLO-320, and SW48) and male (LoVo, SW620, Caco-2, T84, and SW1116) colon cancer cell lines (Table 1 and Table 2) [68,72]. These studies suggested that the expression of PR could be a feature of the tissue rather than a consequence of a malignant process. Moreover, others failed to detect PR in colon cancer cell lines (HT29, HCT116, LoVo, SW480, DLD-1, and Caco-2) [97] or in clinical malignant specimens [78,97], suggesting no role for progesterone in colon neoplasia. 

On the other hand, Tanaka et al. (2008) [95] has reported the expression of PRB but not PRA receptors in normal and neoplastic colonic tissues, as well as in human female and male colon cancer cell lines (HT29 and HCT116). Other investigators showed that PR is highly expressed in colorectal tumor tissues relative to normal colonic tissues, suggesting that PR could play a role in colon carcinogenesis [98,99]. Interestingly, the most recent work by Zhang et al. (2021) [96] showed PR expression in CRC patients correlated inversely with tumor’s size, differentiation, vascular invasion, and advanced clinical stages [100]. The authors also reported that low P4 levels and PR expression in CRC malignant tissues are linked with poor prognosis, suggesting that the hormone could inhibit cancer progression via a PR-mediated mechanism [96].

## 3. Preclinical Studies of Female Sex Steroid Hormones and Their Receptors in CRC

Most of the available preclinical studies on the potential therapeutic effects of female sex steroid hormones and/or their related receptors in CRC are in vitro experiments, and few of them confirmed their results using in vivo animal models of CRC. Notably, the potential therapeutic roles of E2 and/or P4 were mainly investigated in several human male CRC cell lines (Table 1), with few using human female colon cancer cell lines (Table 2). The ages of male patients related to male derivative cell lines ranged between 48 and 73 years (Table 1), and the female patients’ age of female derivative cell lines was 44 years (Table 2).

### 3.1. The Effect of Estrogen and/or Its Receptors

#### 3.1.1. DLD-1 Human Male Colon Cancer Cell Lines 

DLD-1 cells (Table 1) endogenously express ERβ1 isoform and Erα [90,94]. Fiorelli et al. (1999) [90] initially showed that treating DLD-1 cells for 48 h with E2 (10 nM) inhibited the cells proliferation and increased progesterone-specific binding to its receptor (Table 3). E2 treatment at (10 nM) for 24 h or 30 h in DLD-1 cells also inhibited the cells proliferation [40,100], induced cell cycle arrest at the sub-G_1_ phase [100], and triggered apoptosis by increasing cysteine-aspartic-acid-specific protease 3 (caspase-3) production and poly ADP-ribose polymerase (PARP) cleavage [40,91,100,101] (Table 3). Furthermore, the data demonstrated that the actions of E2 in DLD-1 cells were ERβ-dependent, which activated the p38 mitogen-activated protein kinase (p38/MAPK) pathway [40,91,100,101] (Table 3). In contrast, Fiocchetti et al. (2015) [102] also showed that E2 treatment at 10^−8^ M concentration for 24 h upregulated neuroglobin (NGB), which later reallocated into mitochondria, thus increasing oxidative stress, suppressing the release of cytochrome c into the cytosol, and inhibiting cell apoptosis (Table 3). 

A different therapeutic strategy was applied by Bielecki et al. (2011) [103], who investigated the effects of soy isoflavones (genistein, daidzein, and glycitein) at 25 μg/mL for 24 h in DLD-1 cells with overexpression or knocking down of ERβ gene. The soy isoflavones induced cell cycle arrest at the G_2_/M phase by decreasing the expression of cyclin A and cyclin-dependent kinase 4 (CDK4) and increasing the expression of cyclin-dependent kinase inhibitors as p21^(Waf1/Cip1)^ in ERβ-transfected cells [103]. In addition, the soy isoflavones also suppressed the expression of proliferating cell nuclear antigen (PCNA), extracellular-signal-regulated kinase (ERK1/2), alpha serine/threonine protein kinase (AKT), and nuclear factor κB (NF-κB) [103]. Remarkably, the anticancer actions of soy isoflavones were inhibited in DLD-1 cells, lacking ERβ expression, suggesting that the receptor plays a crucial role in mediating the antiproliferative effects of isoflavones in CRC [103]. On the other hand, silencing the expression of ERα effectively inhibited proliferation and colony formation in DLD-1 cells and reduced the tumorigenic capacity in a xenograft mice model [104]. Mechanistically, the deletion of ERα in DLD-1 cells arrested the G_1_ phase of the cell cycle by decreasing the cyclin-dependent kinase 2 (CDK2) activity and hyperphosphorylated state of the retinoblastoma (Rb) protein alongside increasing the expression of p21^Cip1^ and p27^Kip1^ [104].

#### 3.1.2. HCT116 Human Male Colon Cancer Cell Lines 

The HCT116 cells (Table 1) endogenously express ERβ 2–5 isoforms, whilst being negative for ERα expression [73,90,94]. The initial studies in 1999 showed an antiproliferative effect for E2 in HCT116 [90,97], while more recent studies found that treating HCT116 cells with E2 at 1–500 nM for 96 h [94] or at 20 nM for 48 h [95] had no effect on cell proliferation (Table 4). The contradictory results between these studies could be due to the different doses of treatment, cell culture conditions, or methods of proliferation assessment [90,94,95,97].

In the following studies, Hartman et al. (2009) [97] transfected the HCT116 cells with the ERβ gene followed by E2 treatment at 10 nM for 24 h (Table 4), resulting in effective inhibition of cell proliferation, as well as cell cycle arrest at G1 phase by decreasing the expression of myelocytomatosis *oncogene* (MYC) together with increased expression of the p21^(Waf1/Cip1)^ and p27^Kip1^ proteins [97]. Moreover, these anticancer actions were dependent on suppressing the F-box protein (p45^Skp2^) [97]. Similarly, Edvardsson et al. (2011) [105] demonstrated that treating HCT116 cells with E2 following ERβ transfection inhibited cell cycle progression via reducing myelocytomatosis (MYC), myeloblastosis (MYB), and prospero homebox 1 (PROX1) oncogenes, whilst inducing anti-inflammatory responses by decreasing Interleukin-6 (IL-6) expression [105]. 

In contrast, a study by Tu et al. (2012) [106] also found that transfected HCT116 cells with ERβ alone caused differential effects on proliferation, apoptosis, invasion, and metastasis (Table 4). However, raloxifene treatment at 10 µM for 48 h in ERβ-transfected HCT116 cells synergistically inhibited cell proliferation, induced apoptosis, reduced invasion, and halted cell migration compared with ERβ alone and/or raloxifene alone [106]. The authors confirmed their findings in a CRC xenograft model [106]. Raloxifene is an ER modulator that either exhibits estrogenic or antiestrogenic effects depending on tissue types [106].

Recent work by Topi et al. (2020) [88] has also demonstrated that an ERβ-selective agonist (ERB-041) induced anticancer activities in HCT116 cells by inhibiting cell proliferation, provoking apoptosis through the action of caspase-3, and decreasing cell migration (Table 4). Mechanistically, this action occurred by increasing the expression of ERβ and decreasing the nuclear expression of β-catenin, cysteinyl leukotriene receptor 2 (CysT_2_R), and 15-hydroxyprostandlin dehydrogenase (15-PGDH), which consequently reduced the expression levels of cyclin D1 and c-MYC [88]. The authors also used a zebrafish xenograft model, and treatment with ERB-041 alone for 48 h reduced distant metastasis compared with the untreated control group [88].

Another study by Bernatchez et al. (2013) [104] reported marked in vitro inhibitions in cell proliferation and colony formation following silencing the expression of ERα subtype in HCT116, as well as significant reductions in tumorigenic capacity using s xenograft mouse model injected with HCT116 cells (Table 4). The ERα-depleted HCT116 cells also showed cell cycle arrest at G_1_ and S phases following decreases in CDK2 activity and hyperphosphorylated state of the Rb protein with concurrent increases in p21^Cip1^ and p27^Kip1^ proteins [104]. ERα inhibition also reduced the expression of essential genes related to glycolysis, tricarboxylic acid cycle, lipid synthesis, glucose incorporation, and glucose-mediated lipogenesis [104]. In agreement, Zhou et al. (2018) [107] reported that suppression of ERRα in HCT116 cells by the FDA-approved ERRα activity inhibitor simvastatin, at 10 μM for 48 h, inhibited cell proliferation and colony formation by reducing the expression of c-MYC and cyclin D1 proteins (Table 4). In addition, the researchers proclaimed that simvastatin also enhanced the cytotoxicity of trametinib, an MEK inhibitor (50 nM), when used in combination for 48 h [107]. Consequently, they also showed that the combination of both drugs had synergistically inhibited cell proliferation and survival in a xenograft mouse model following subcutaneously implanting HCT116 cells transfected with ERRα genes [107]. Altogether, the findings of earlier studies suggest that ERRα could have oncogenic activities in CRC cells and suppressing its expression alone or combined with treatment with MEK inhibitor could represent promising efficient therapeutic strategies against CRC [107].

#### 3.1.3. SW480 and SW620 Human Male Colon Cancer Cell Lines

The SW480 cells were derived from a primary tumor collected from a 51-year-old male patient diagnosed with Dukes’ type B CRC, whereas the SW620 cells were generated from lymph node metastases (Dukes’ type C) from the same patient of SW480 cells [89] (Table 1). Regarding the endogenous ERs expression, SW480 cells express ERβ and lack ERα [94]. In contrast, the SW620 cells express both ERα and ERβ [73]. The current studies found that 10 nM E2 treatment for 24 h following insertion of ERβ in the SW480 cells inhibited cells proliferation [97], arrested cell cycle at G_0_/G_1_ phase [97,105] and induced anti-inflammatory response via reducing the IL-6 expression [105] (Table 5). Mechanistically, inhibition of proliferation and/or cell cycle arrest was induced by decreases in MYC [96,108], MYB, and PROX1 [105] oncogenes; additionally, inhibition caused increases in p21^(Waf1/Cip1)^ and p27^Kip1^ expression [97]. In 2013, Edvardsson et al. [108] also demonstrated that the re-expression of ERβ with E2 treatment regulated the microRNA (miRNA) pool in SW480 cells, and ERβ-induced downregulation of MYC oncogene strongly suppressed the oncogenic miR-17-92 cluster and miR-200a/b, which then promoted cell death upon DNA damage in ERβ-expressing SW480 cells [108]. Furthermore, they also determined that the suppression of miR-200a/b decreased the expression levels of E-cadherin, which then impaired cell migration in the ERβ-expressing SW480 cells [108]. These studies demonstrated that exogenously re-expressed ERβ in combination with E2 treatment could act as an anticancer strategy in SW480 cells [97,105,108]. On the other hand, inserting the ERβ gene in the SW620 metastatic CRC cells showed no effects on MYC and miRNAs [108], suggesting that E2 treatment following ERβ activation could be more beneficial for preventing and/or treatment of early stage, but not advanced stages, colon cancers.

In contrast, Nguyen-Vu et al. (2016) [109] reported no effects following E2 treatment in the SW480 (Table 5) and SW620 (Table 6) cells, suggesting the E2-induced anticancer activities could be dependent on the expression status of ERβ in these cell lines [109]. Moreover, the authors found that ERβ transfection in the SW480 and SW620 cells increased the levels of miR-205 and suppressed the expression of PROX1 oncogene, which subsequently inhibited cell growth, arrested cell cycle at the G_0_ phase, and increased cell adhesion [109] Interestingly, ERβ re-expression along with high expression levels of miR-205 also reduced invasion and/or metastatic potential, particularly in SW620 metastatic colon cancer cells [109] (Table 6).

Another promising treatment strategy has also been suggested by Zhou et al. (2018) [107], who transfected the SW480 cells while with ERRα isoform followed by simvastatin treatment (ERRα activity inhibitor) at 10 μM for 48 h; which effectively reduced cell proliferation and colony formation via decreasing the expression of c-MYC and cyclin D1 [107]. Simvastatin also enhanced the cytotoxicity of trametinib (MEK inhibitor) at 50 nM when used in combination for 48 h in the SW480 cells [107] (Table 5). Collectively, there is a compelling need to explore the potential therapeutic effects of E2 and its receptors in the SW620 metastatic cells, since only a few studies are available (Table 6).

#### 3.1.4. LoVo Human Male Colon Cancer Cell Lines

LoVo cells (Table 1) endogenously express ERβ and lack ERα [95,98]. In contrast, Qui et al. (2002) [73] found that LoVo cells expressed with both ERβ6 and Erα. Among the current studies (Table 7), only one study showed that E2 treatment had no effects on the proliferation of LoVo cells [94]. The remaining works found that E2 treatment at 10^−8^–10^−6^ M doses [110] or 10 nM [90] for 48 h efficiently reduced the proliferation of LoVo cells (Table 7). Lointier et al. (1992) [110] showed that progesterone and androgen hormones were less effective than E2 treatment. Consequently, a study by Hsu et al. explored the functions of the E2 and its receptors and determined the potential molecular targets of the E2-ERβ complex in LoVo cells (Table 7). Following transfecting LoVo cells with ERβ [111] or ERα [112], treatment with E2 at 10^–8^ M for 12–16 h effectively inhibited cell proliferation, arrested the cell cycle at G_1_ and S phases via increasing the expression of p21^(Waf1/Cip1)^ and p27^Kip1^, decreased β-catenin, cyclin D1/E and Rb gene and protein expression, and induced apoptosis and DNA fragmentation through activating the caspases-8, -9, and/or 3 [111,112] (Table 7). Remarkably, the anticancer action of ERβ with E2 was mediated through upregulating the expression of p53 [111], whereas ERα with E2 was propagated by increasing the expression of TNF-*α* signaling [112] in LoVo cells (Table 7). 

Later studies focused on exploring the effect of E2 treatment on migration-related factors, such as urokinase plasminogen activator (uPA), tissue plasminogen activator (tPA), and matrix metallopeptidases (MMPs), in LoVo cells [113,114,115,116] (Table 7). Interestingly, E2 treatment impeded the migration of LoVo cells and downregulate the expression of its related proteins (u-PA, t-PA, MMP-9, and MMP-2/9) through modulating the signaling pathways of AKT and ERK1/2 [113], JNK1/2 [114], p38 MAPK [115], and p53 [116] (Table 7). Additionally, the E2-ERs complex (ERα and ERβ) mediation also suppressed migration-related factors in LoVo cells [115] and was associated with inhibition of cell proliferation by modulating the expression of cell-cycle-regulating factors, including cyclin A, D1, and E [115] (Table 7). Taken together, these findings suggested that E2-ERs, particularly ERβ, could be a promising strategy for the treatment of advanced cases of CRC male patients.

#### 3.1.5. HCT8 Human Male Colon Cancer Cell Lines

HCT8 cells (Table 1) endogenously express ERβ 1–5 isoforms and lack ERα [90]. Earlier work showed that treating the HCT8 cells with E2 at 10 nM for 48 h had no effects on cell proliferation [90] (Table 8). In contrast, Martineti et al. (2005) [117] reported that E2 at 10 nM for 24 h following ERβ transfection in HCT8 cells inhibited cell proliferation, and induced cell cycle at G_1_ and S phases via reducing cyclin E and increasing p21^Waf1/Cip1^ expression (Table 8). An explanation for the contradictory results reported could be the different treatment conditions and overexpression of ERβ by gene insertion.

#### 3.1.6. COLO205 Human Male Colon Cancer Cell Lines

COLO205 cells (Table 1) were reported to express ERβ1, 2, 5, and 6 isoforms, whilst lacking endogenous ERα expression [73]. Qui et al. (2002) [77] found that E2 treatments for 48 h with doses equal to those attained in females receiving HRT for postmenopausal symptoms (10^−12^, 10^−10^, 10^−7^ M) induced apoptosis and DNA fragmentation in COLO205 cells. Hence, they proposed the anticancer actions of E2 in CRC were mediated by ERβ-dependent pathways [73]. E2 treatment also inhibited proliferation and induced apoptosis in COLO205 cells by upregulating DNA-damage-inducible protein 153 (GADD153) and suppressing the expression of RAS, PKB, AKT, MYC, VEGF, and others [118] (Table 9). In agreement with these studies, Wilkins et al., 2010 [119] also showed that E2 treatments, at 10^−11^–10^−7^ M for 24 h, reduced the numbers of viable COLO205 cells and induced late apoptosis and DNA fragmentation via decreasing the expression of c-MYB oncogene and anti-apoptotic BCL-2 proteins [119] (Table 9).

#### 3.1.7. HT29 Human Female Colon Cancer Cell Lines

HT29 cells (Table 2) were reported to express ERβ1 isoform, but not ERα [72,94]. In contrast, another study showed that HT29 cells expressed both estrogen receptors (ERβ and ERα) [73]. Earlier studies disclosed that E2 treatments at 1–500 nM for 96 h [94] or at 20 nM for 48 h [95] did not inhibit proliferation in HT29 cells (Table 10). Although the reasons behind the lack of antiproliferative effect in HT29 cells are not entirely understood, a possible explication could be due to the lower ERβ gene expression in these cells compared with HCT116 and SW480 male CRC cells lines [94]. In addition, the expression of ERα in HT29 cells could underly the proliferative effects of E2 [73], since the receptor contributes significant roles to cell growth. In support to the previous suggestion, treating human female CRC cell lines (HT29 and COLO320) with the ERα inhibitor tamoxifen significantly inhibiting proliferation [94]. 

Although Hartman et al. [97] also reported that ERβ transfection in HT29 cells followed by 10 nM E2 treatment for 24 h did not inhibit cell proliferation, it induced cell cycle arrest at G_1_ phase by decreasing the expression of c-MYC, F-box, and p45^Skp2^ proteins, with concurrent increases in p21^(Waf1/Cip1)^ and p27^Kip1^ expression. Likewise, more recent studies that utilized a similar treatment strategy in HT29 cells showed cell cycle arrest with reduced expression of MYC, MYB, and PROX1 oncogenes, as well as the proinflammatory cytokine IL-6 [105]. Moreover, Nguyen-Vu et al. (2016) [109] found that the transfection of HT29 cells with ERβ directly increased the levels of miR-205 and suppressed the expression of PROX1 oncogene, which subsequently inhibited cell proliferation, induced G_0_ phase arrest, increased cell adhesion, and reduced cell invasion and metastasis. Recently, Topi et al. (2020) [88] used a zebrafish xenograft model that was injected with HT29 cells with ERβ-selective agonist (ERB-041) monotherapy for 48 h, and their results showed reductions in distant metastasis compared with the control group (Table 10) [88]. 

Other research has transfected the HT29 cells with ERα46 form followed by treatment with E2 at 10^−8^ mol/L for 48 h, which subsequently inhibited the cells proliferation, arrested the cell cycle at the G_0_/G_1_ phase, and induced apoptosis [77]. Indeed, ERα could produce different actions in the colon carcinogenesis, dependent on the existence of several splice forms of the receptor; among these, ERα46 has been shown to induce antiproliferative and apoptotic activities, while the shorter variant, ERα36, was associated with tumor progression in the colon [77]. Furthermore, the HT29 cells expressed low levels of ERα46 mRNA as determined by RT-qPCR [77]. Clinically, the expression of ERα46 mRNA levels is low in human colorectal tumor tissues compared with the normal adjacent colorectal tissues [77].

Bernatchez et al. (2013) [104] used another strategy and silenced the expression of ERRα in HT29 cells, showing that it could delay the cells’ transition from the G_0_/G_1_ phase to the S phase via decreasing the expression of CDK2 activity and hyperphosphorylated state of the Rb protein, along with increasing the expression of p21^(Waf1/Cip1)^ and p27^Kip1^. They suggested that silencing the expression of ERRα could be a promising therapeutic strategy for female CRC cells [104]. In fact, ERRα has appeared as a transcriptional metabolic regulator, promoting many processes linked with cancer development and progression [120]. High expression of ERRα is correlated with poor prognosis for the cancers of the ovary, breast, prostate, endometrium, and colon [120]. Overall, the available data suggest and re-expression or hyperactivation of ERβ and/or ERα46, whereas the inhibition/blocking of ERRα could provide promising effective therapeutic strategies for treating CRC female patients.

### 3.2. The Effect of Progesterone and/or Its Receptors

The available data on the roles of natural progesterone (P4) and synthetic progestins and/or their receptors in colon carcinogenesis is limited compared with estrogen and its receptors. Although synthetic progestins are bioidentical to endogenous P4, they differ in their chemical structures [121]. One of the most used synthetic progestins in OC and HRT is medroxyprogesterone acetate (MPA) [28,121,122]. Although physiologic MPA has high affinity to progesterone receptors, the drug could also bind other steroid receptors, including glucocorticoids, androgen, and mineralocorticoid receptors [123]. MPA has been successfully applied for treating advanced breast, prostate, and endometrial cancers [122]. The effects of MPA and/or P4 were studied in several male CRC cell lines (HCT116, COLO205, DLD-1, LoVo, SW480, SW620) and a female colon cancer cell line (HT29), as well as in a few in vivo experimental models (Table 11).

Regarding the expression of PR in CRC cell lines, most male cells (HCT116, DLD-1, SW480) were negative for endogenous PR, whereas the SW620 and LoVo metastatic cells showed differential expression profiles of PR (PR^−^/low PR/PR^+^) (Table 1). On the other hand, PR was found to be naturally expressed by the male COLO205 and female HT29 cells (Table 1 and Table 2).

Tanaka et al. (2008) [95] treated in vitro human HT29 female and HC116 male colon cancer cell lines with MPA at 20 nM for 48 h following transfecting the PR gene, and the results revealed potent antiproliferative effects and cell cycle arrest at the G_0_/G_1_ phase by decreasing the expression of cyclin E and increasing the expression of p21^WAF1/CIP1^ (Table 11). In contrast, natural P4 treatment showed no effects on cell proliferation in PR-transfected HT29 and HCT116 cells [95] (Table 11).

Moreover, another two studies suggested that the anticancer activity of P4 could be enhanced by increasing endogenous expression of PR in colon cancer cells [93,124]. In this regard, the researchers showed that decreased growth in PR^+^ COLO205 male [93] and PR^+^ HT29 female [124] colon cancer cells following treatment with natural progesterone. P4 also induced apoptosis via increasing caspase-8 activity [124] (Table 11). The contradictory effects of MPA and P4 in CRC treatment could be due to the chemical differences, which could produce different actions at the cellular level, binding potencies to PR in colon cancers, treatment conditions, and/or different types of colon cancer cell lines.

On the other hand, Heijmans et al. (2011) [97] found that both MPA and P4 monotherapies had no effects on cell proliferation in HT29, HCT116, SW480, DLD-1, and LoVo CRC cell lines (Table 11). This study also failed to detect significant effects for MPA treatment (25 mg/day; 90 days) by subcutaneous implants on colon tumorigenesis in female rodents [96], which agrees with another recent in vivo study demonstrated no anticancer effects for P4 single treatment in Sprague–Dawley female rats with CRC [125] (Table 11).

In contrast, another in vivo study by Meijer et al. (2018) [126] has shown that MPA monotherapy reduced colon tumorigenesis in a postmenopausal mouse model, but not in fertile female mice, suggesting that synthetic progestin could be an effective chemopreventive only in postmenopausal women. Consistently, more recent research also demonstrated that P4, as monotherapy-inhibited cell proliferation, arrested the cell cycle at the G_2_/M phase, and induced apoptosis in LoVo and SW620 metastatic male colon cancer cells; in addition, it was found to inhibit tumor growth in a xenograft CRC model with implanted SW620 cells [96]. Furthermore, apoptosis was induced by decreasing the expression of BCL2 and increasing the expression of BAX, which consequently activated caspase-3 in the treated cells [96]. In addition, the P4 therapy was associated with increased activity of the JNK pathway via upregulating the expression of antiproliferative and DNA-damage-inducible protein α (GADD45α) in colon cancers [96] (Table 11).

Collectively, the results of the different studies (Table 11) demonstrated the tumoricidal efficacy of MPA and/or P4 in CRC, which could support the use of the natural or synthetic forms of the hormone in the treatment of this common malignancy.

**Table 11:** Summary of studies that measured the effects of synthetic progesterone (medroxyprogesterone (MPA))/natural progesterone (P4) and/or its related receptor in CRC in vitro cells/in vivo models in chronological order.

CRC Cells/Models	Treatment Strategy		Potential Actions	Suggested Mechanisms	References
	Gene Insertion/Deletion	Exogenous Treatment			
In Vitro HCT116, HT29	Transfected with PR	MPA (20 nM) for 48 h	Inhibited cell proliferation Arrested cell cycle at G_0_/G_1_ phase	↓ Cyclin E expression ↑ p21^WAF1/CIP1^ expression	Tanaka et al., 2008 [95]
	Transfected with PR	P4 (20 nM) for 48 h	# No effect on proliferation	-	Tanaka et al., 2008 [95]
In Vitro COLO 205	PR+	P4 (50 nM) for 4 days	Inhibited cell proliferation	-	Kuo et al., 2016 [93]
In Vitro HT29	PR+	P4 (0.01 mg/mL)	Inhibited cell proliferation Induced apoptosis	↑ Caspase-8 activity	Sirati Moghaddam et al., 2017 [124]
In Vitro DLD-1, HCT116, SW480, LoVo, HT29	-	MPA (0.2, 2, 20, 200 ng mL^−1^) for 48 h	# No effect on proliferation	-	Heijmans et al., 2011 [97]
	-	P4 (0.2, 2, 20, 200 ng mL^−1^) for 48 h	# No effect on proliferation	-	
In Vivo Female rodent models	-	MPA (25 mg) implanted subcutaneously in the neck in a 90-day	# No effect on colon tumorigenesis	-	
In Vivo Female mice model at the postmenopausal state.	-	MPA (7.5 mg) in 90 days	Reduced tumorigenesis	-	Meijer et al., 2018 [126]
In Vivo Sprague–Dawley female rats with CRC	-	P4 (10 mg/kg) Injected the rats twice a week, observed at 84 h after the last injection.	# No effect on proliferation	-	Sasso et al., 2019 [125]
In Vitro LoVo, SW620	-	P4 (250 µM) for 48 h	Inhibited cell proliferation Arrested cell cycle at G_2_/M phase Induced apoptosis	↓ BCL-2 antiapoptotic protein ↑ BAX proapoptotic protein ↑ Caspase-3 activity ↑ JNK activity ↑ GADD45α expression	Zhang et al., 2021 [96]
In Vivo Xenograft CRC Model (Implanted with SW620 cells into the dorsal subcutaneous tissue of mice to study tumor growth)			Inhibited tumor growth Induced apoptosis	↑ JNK activity ↑ GADD45α expression	

Symbols: ↑—increase; ↓—decrease; #—no effect. Grey shading indicates in vivo experiment.

### 3.3. The Combination Effect of Estrogen and Progesterone

Experimental studies on the combinatory effects of estrogen and progesterone in CRC are very limited, and only one in vitro [95] and another in vivo study are available at the present [125] (Table 12). Tanaka et al. (2008) [95] found that synthetic progestins, such as MPA at 20 nM both as monotherapy and in combination with E2, inhibited cell proliferation of human female (HT29) and male (HCT116) colon cancer cells, whereas single hormonal treatments showed no anticancer effects in either cell line [95]. Likewise, the in vivo study by Sasso et al. (2019) [125] found that individual treatments with E2 or P4 had no effects on cell proliferation and apoptosis in colonic tumors induced in Sprague–Dawley female rats [125]. In contrast, the combination of E2 (60 μg/kg) and P4 (10 mg/kg) induced enhanced reductions in cell proliferation and promoted apoptosis by increasing the expression of cleaved caspase-8, caspase-3, and PARP in malignant tissues [125]. The combination treatment was also associated with higher expression of ERβ in colonic tumors [126], suggesting that the anticancer actions of E2 and P4 co-therapy could be dependent on the activation of the ERβ receptor.

## 4. Key Findings of Preclinical Studies

The current knowledge about the therapeutic strategies and potential actions of female sex steroid hormones (estrogen and progesterone) in CRC is greatly based on preclinical studies. Therefore, this review can provide a groundwork that will encourage more detailed research at the clinical level, considering the most promising therapeutic strategies of female sex steroids in the treatment of either males or females with CRC.

The most promising therapeutic strategies were E2 exogenous treatment and re-activation of the ERβ subtype, either alone or in combination, or inhibition of the ERα subtype alone (Table 3, Table 4, Table 5, Table 6, Table 7, Table 8, Table 9 and Table 10). These strategies have been effectively exerted multi anticancer effects including antiproliferative, proapoptotic, antiangiogenetic, and/or antimigratory effects, both in in vitro human colon cancer cell lines (mainly for male cells that derived from advanced stages of CRC) and in vivo CRC models. Remarkably, E2 treatment could produce its anticancer actions by mediating the ERβ in CRC cells. The anticancer activities of those strategies have occurred by regulating different genes and/or protein expression, including tumor suppressor genes such as p53 and Rb; oncogenes such as MYC, MYB, and PROX1; cell cycle regulators such as p21, p27, and cyclin A/D/E; caspase cascades such as caspase-9 and -3; migration-related factors such as uPA, uPA, MMP-2/9; and signaling pathways such as p38/MAPK, AKT, ERK1/2, JNK1/2, which are commonly linked with onset and progression of CRC. The in vitro experiments were limited in female colon cancer cell lines, which were less responsive to some therapeutic strategies, particularly E2 monotherapy, than in vitro male CRC cells. This could be due to the overexpression of the ERα subtype.

The other possible therapeutic strategy was the progesterone exogenous treatment (natural or synthetic progesterone) (Table 11), which was also associated with promising tumoricidal actions mediated via PR, while the available studies on progesterone are still limited. In addition, there were some inconsistent findings between the effect of natural progesterone (P4) and synthetic progesterone (MPA), and this could be due to the different nature of progesterone compounds that were used, different patterns of PR expression, or different types of CRC cell lines/models.

Nevertheless, the combination of E2 with P4 (Table 12) showed enhanced anticancer activities compared with their monotherapies in male–female cell lines and animals, which could be also another promising therapeutic approach for CRC treatment, but the studies were very limited, and the action mechanisms remain to be further elucidated. Together, the studies suggested that female sex steroid hormones could provide a novel and effective therapeutic method against CRC.

## 5. Limitations of Female Sex Steroid Hormones in Cancer Treatments

Female sex steroid hormones can exert pro- or antitumorigenic actions depending on the origin of the cancer type, the endogenous expression patterns of their receptors in the tissue, and the stage of cancer [127,128]. In this regard, the actions of E2 in CRC have shown to be receptor-dependent actions; ERα exerts oncogenic actions, whilst ERβ exerts antitumorigenic actions [129]. In this context, E2 therapy promoted cell cycle progression and cell survival via ERα-mediated mechanisms, while inducing apoptosis via activating caspase-3 by ERβ in DLD1 CRC cells [130]. Moreover, ERα and ERβ presented opposing effects on cell cycle progression in HeLa human cervical cancer cells via increasing and decreasing the gene expression of cyclin D1, respectively [131]. Paterni et al. (2014) [127] reported that selective stimulation of ERβ could be as effective as a preventive and/or therapeutic approach against some types of cancers, such as CRC. Because estrogens have been shown to inhibit proliferation and induce apoptosis in CRC, these protumorigenic actions are believed to be mediated through the ERβ, which is a predominant estrogen receptor subtype in the colon [73,132]. The expression of ERβ is decreased during colon carcinogenesis, as discussed in Section 2.1 [38,71,79,128].

Notably, genetic studies have also found that both ERβ and PR pathways interacted together in CRC, and PR antitumorigenic actions were also dependent on the activities of ERβ in malignant tissues [133]. Furthermore, concomitant E2 and P4 dual therapy markedly increased the protein expression of ERβ, PR, and caspase-3 alongside cell apoptosis, whereas it downregulated cell proliferation markers, compared with single hormonal therapies in an ovariectomized female rat model of CRC [125]. However, none of the prior studies measured the potential antitumorigenic effects of E2 and P4 sequential treatment in CRC, although this protocol mimics normal endocrinology in reproductive-age females. Most current studies suggested that E2 and/or P4 could be alternative therapeutic strategies against CRC, and their effectiveness could be dependent on the expression profiles of ERs and PR in colon cancer tissues [125,133,134,135].

Indeed, the molecular pathways mediated via ERs and PR interact together, and the activation of one receptor could influence the activities of the other in neoplastic diseases [136]. In this context also, the activation of ERβ [134] and PR [135] inhibited the growth of breast cancer cells by suppressing the ERα-induced oncogenic actions. In contrast to colon cancer, the estrogens (either endogenous or exogenous) have been shown to induce cell proliferation and tumor development in the breast and uterus, and these protumorigenic effects are supposed to be mediated via the ERα, which is predominant in these tissues [132]. In fact, female sex steroid hormones and their receptors play a vital role in breast cancer development and progression [137]. Almost 70% of breast cancers are hormone-receptor-positive cancers (ER^+^ and/or PR^+^), which are correlated to cancer cell growth, progression, and spread [137]. The PR modulates ERα action and an upregulated target gene of ER, and its expression is dependent on estrogen in breast cancer [137]. Thus, ERα-antagonists have currently proved to be effective agents for reducing/inhibiting cell proliferation in hormone-sensitive breast cancer [137].

Normally, ERα is found mainly in the mammary gland, uterus, ovary (thecal cells), prostate (stroma), male reproductive organs (testes and epididymis), bone, liver, and adipose tissue [127]. While ERβ is present mainly in the colon, ovary (granulosa cells), prostate (epithelium), bladder, adipose tissue, and immune system [127], the alpha subtype has a more prominent role on the mammary gland and uterus, and induces cell proliferation in tissues including breast and uterus. The beta subtype generally counteracts the ERα-induced cell hyperproliferation in breast and uterus tissues [127]. The actions of estrogens are modulated by the ERs subtypes (ERα and ERβ), and estrogen-mediated ERs subtypes can exert beneficial effects in some types of cancers, such as colon cancer; however, they can have adverse and severe effects in breast and uterus tissues [127]. Thus, more profound knowledge of the various biological activities of ERα or ERβ in different tissues would help in the decision of which receptor subtype is the best to use for the management or cure of cancers [127].

Deli et al. (2019) [138]. have also classified the oncologic risk (recurrence and progression) of hormonal replacement therapy (HRT) in cancers for females, as follows: (a) HRT is advantageous for colon cancer, endometrial cancer type I, cervical adenocarcinoma, hepatocellular cancer, hematologic malignancies, and local cutaneous malignant melanoma; (b) HRT is neutral and thus it should not be denied for BRCA 1/2 mutation carriers without cancer, endometrial cancer type II, uterine carcinosarcoma, and adenosarcoma, certain types of ovarian cancer, cervical cancer, kidney cancer, pancreatic cancer, and thyroid cancer; (c) HRT is relatively contraindicated, and thorough, individualized decision making is essential for certain types of ovarian tumors, brain tumors, leiomyosarcoma, advanced metastatic malignant melanoma, lung cancer, gastric cancer, and bladder cancer; (d) HRT is disadvantageous and harmful for hormone-dependent cancers, such as breast cancer, endometrial stroma sarcoma, hormone-receptor-positive (ER^+^/PR^+^) gastric and bladder cancer, meningioma, and glioma [138].

Overall, it is essential to consider the hormonal factors during cancer occurrence before using any hormonal therapy, as well as evaluate the exposure patterns to endogenous sex hormones, such as age at menarche and menopause, years of periods/fertility, number of pregnancies and births, duration of breastfeeding, and other related factors; iin addition, it is important to evaluate the exposure to exogenous hormones, such as HRT, OC, or fertility treatments [128,138].

## 6. Conclusions

The roles of female sex steroid hormones (estrogen and progesterone) and their nuclear receptors in CRC represent an attractive avenue for developing novel efficient alternative hormonal therapies. Most of the currently available data suggest that E2 could induce cancer progression via ERα whilst promoting ERβ-mediated anticancer activities. Possible approaches may involve overexpression and/or selectively stimulating ERβ, with and without selective inhibition of ERα activities in male and female CRC patients. Similarly, natural and synthetic progesterone showed promising tumoricidal actions that were mediated via PR in male and female experimental models. Nevertheless, the combination of E2 with P4 appears to be a more effective strategy, relative to individual hormonal therapy, both in vitro and in vivo. Hence, additional research is needed to explore the therapeutic values of E2 and P4 combined therapy, with and without androgen deprivation therapy, in colon cancer.

## Figures and Tables

**Table 1:** Expression of female sex steroids nuclear receptors in human male colon cancer cell lines.

Cell Line	ATCC No	Isolated from	Age	Malignancy Stage and/or Grade	Endogenous Expression of ERs/PRs	References
**DLD-1**	ATCC [89] [CCL-221™]	Primary tumor	Adult (Unknown age)	Colorectal adenocarcinoma. Dukes’ type C	ERα^−^ and ERβ1^+^	Fiorelli et al., 1999 [90]
					ERβ1^+^	Galluzzo et al., 2007 [91]
					PRA^−^ and PRB^−^	Heijmans et al., 2011 [92]
					PR^−^	Kuo et al., 2016 [93]
**HCT116**	ATCC [89] [CCL-247™]	Primary tumor	48 Y	Colorectal Carcinoma. Dukes’ type D	ERα^−^ and ERβ1-5^+^	Fiorelli et al., 1999 [90]
					ERα^−^ and ERβ^+^	Arai et al., 2000 [94]
					ERβ1^+^, 2^+^, 5^+^, 6^+^	Qui et al., 2002 [73]
					PRA^−^ and PRB^+^	Tanaka et al., 2008 [95]
					PRA^−^ and PRB^−^	Heijmans et al., 2011 [92]
					PR^−^	Kuo et al., 2016 [93]
**SW480**	ATCC [89] [CCL-228™]	Primary tumor	51 Y	Colorectal adenocarcinoma. Dukes’ type B (3rd–4th grade)	ERα^−^ and ERβ^+^	Arai et al., 2000 [94]
					PRA^−^ and PRB^−^	Heijmans et al., 2011 [92]
**SW620**	ATCC [89] [CCL-227™]	From lymph node metastasis from the same patient of SW480	51 Y	Colorectal adenocarcinoma. Dukes’ type C	ERα^+^ and ERβ6^+^	Qui et al., 2002 [73]
					Low PR	Hendrickse et al., 1993 [68]
					Low PR	Zhang et al., 2021 [96]
**LoVo**	ATCC [89] [CCL-229™]	Metastatic tumor nodule in the left supervascular region	56 Y	Colorectal adenocarcinoma. Dukes’ type C (grade IV)	ERα^−^ and ERβ1^+^	Fiorelli et al., 1999 [90]
					ERα^−^ and ERβ^+^	Arai et al., 2000 [94]
					ERα^+^ and ERβ6^+^	Qui et al., 2002 [73]
					Low PR	Hendrickse et al., 1993 [68]
					PRA^−^ and PRB^−^	Heijmans et al., 2011 [92]
					PR^+^	Kuo et al., 2016 [93]
					High PR^+^	Zhang et al., 2021 [96]
**HCT-8**	ATCC [89] [CCL-244™]	-	67 Y	Colorectal adenocarcinoma	ERα^−^ and ERβ1-5^+^	Fiorelli et al., 1999 [90]
**COLO 205**	ATCC [89] [CCL-222™]	-	70 Y	Colorectal adenocarcinoma. Dukes’ type D	ERα^−^ and ERβ1, 2, 5, 6^+^	Qui et al., 2002 [73]
					PR^+^	Kuo et al., 2016 [93]
**Caco-2**	ATCC [89] [HTB-37™]	-	72 Y	Colorectal adenocarcinoma	ERα^+^ and ERβ6^+^	Qui et al., 2002 [73]
					ERα^−^ and ERβ1, 2, 5^+^	Campbell Thompson et al., 2001 [72]
					Low PR	
					PRA^−^ and PRB^−^	Heijmans et al., 2011 [92]
**SW1116**	ATCC [89] [CCL-233™]	-	73 Y	Colorectal adenocarcinoma. Dukes’ type A	ERα^−^ and ERβ2,5^+^	Campbell Thompson et al., 2001 [72]

**Table 2:** Expression of female sex steroids nuclear receptors in human female colon cancer cell lines.

Cell Line	ATCC No	Isolated from	Age	Malignancy Stage and/or Grade	Endogenous Expression of ERs/PRs	References
**SW 48**	ATCC [89] [CCL-231™]	-	82 Days	Colorectal Adenocarcinoma. Dukes’ type C (Grade IV)	ERα^−^ and ERβ2,5^+^	Campbell Thompson et al., 2001 [72]
					Low PR	
**SW403**	ATCC [89] [CCL-230™]	Primary tumor	(Unknown age)	Colorectal Adenocarcinoma. Dukes’ type C (Grade III)	-	-
**HT29**	ATCC [89] [HTB-38™]	Primary tumor	44 Y	Colorectal Adenocarcinoma. Dukes’ type C	ERα^−^ and ERβ^+^	Arai et al., 2000 [94]
					ERα^+^ and ERβ6^+^	Qui et al., 2002 [73]
					ERα^−^ and ERβ^+^	Campbell Thompson et al., 2001 [72]
					Low PR	
					Low PR	Hendrickse et al., 1993 [68]
					PRA^−^ and PRB^+^	Tanaka et al., 2008 [95]
					PRA^−^ and PRB^−^	Heijmans et al., 2011 [92]
					PR^+^	Kuo et al., 2016 [93]
**COLO 320**	ATCC [89] [CCL-220™]	Primary tumor	55 Y	Colorectal Adenocarcinoma.	ERα^−^ and ERβ^+^	Arai et al., 2000 [94]
					Low PR	Hendrickse et al., 1993 [68]

**Table 3:** Summary of studies that measured the effects of estrogen and/or its related receptors in DLDL-1 cells^(ERα^^−^^& ERβ+)^ in chronological order.

Treatment Strategy		Potential Actions	Suggested Mechanisms	References
Gene Insertion/Deletion	Exogenous Treatment			
-	E2 (10 nM) for 48 h	Inhibited cell proliferation Increased the progesterone-specific binding	-	Fiorelli et al., 1999 [90]
-	E2 (10 nM) for 24–30 h	Inhibited cell proliferation Arrested cell cycle at sub-G_1_ phase Induced apoptosis	↑ p38 phosphorylation ↑ Caspase-3 activation ↑ PARP cleavage	Marino et al., 2006 [40]
-	E2 (10 nM) for 24 h	-	↑ ERβ levels via the palmitoylation-dependent persistentp38/MAPK activation.	Caiazza et al., 2007 [101]
-	E2 (10 nM) for 30 h	Induced apoptosis	↑ p38 phosphorylation ↑ Caspase-3 activation ↑ PARP cleavage	Galluzzo et al., 2007 [91]
-	E2 (10^−8^ M) for 24 h	Inhibited cell proliferation Induced apoptosis	↑ p38 phosphorylation ↑ ERβ expression ↑ Caspase-3 activation ↑ PARP cleavage	Bolli et al., 2010 [100]
Transfected with ERβ gene.	Soy isoflavones (25 μg/mL) for 24 h	Inhibited cell proliferation Arrested cell cycle at G_2_/M phase	↓ PCNA, ERK-1/2, AKT, NF-κB expression ↓ Cyclin A expression ↓ CDK-4 expression ↑ p21^(Waf1/Cip1)^ expression	Bielecki et al., 2011 [103]
Silenced ERβ gene		# No effect on cell proliferation # No effect on cell cycle progression	# No effect on all molecular events that were seen in transfected cells	
Silenced/depleted of endogenous ERα	-	Inhibited cell proliferation Arrested cell cycle at the G_1-_S phase Inhibited colony formation	↓ CDK2 activity expression ↓ Hyperphosphorylated state of Rb ↑ p21^(Waf1/Cip1)^ expression ↑ p27^Kip1^ expression	Bernatchez et al., 2013 [104]
Silenced/depleted of endogenous ERα in in vivo (mice) Xenograft model. Injected with DLD-1 CRC cells	-	After 32 days after inoculation: Reduced the tumorigenic capacity	-	
-	E2 (10^−8^ M) for 24 h	Induced NGB upregulation Inhibited the proapoptotic induction	↑ Oxidative stress ↓ Cytochrome c	Fiocchetti et al., 2015 [102]

Symbols: ↑—increase; ↓—decrease; #—no effect; grey shading indicates in vivo experiment.

**Table 4:** Summary of studies that measured the effects of estrogen and/or its related receptors in HCT116 cells^(ERα^^−^^& ERβ+)^ in chronological order.

Treatment Strategy		Potential Actions	Suggested Mechanisms	References
Gene Insertion/Deletion	Exogenous Treatment			
-	E2 (10 nM) for 48 h	Inhibited cell proliferation Increased the progesterone-specific binding	-	Fiorelli et al., 1999 [90]
-	E2 (1 to 500 nM) for 96 h	# No effect on cell proliferation	-	Arai et al., 2000 [94]
-	E2 (20 nM) for 48 h	# No effect on cell proliferation	-	Tanaka et al., 2008 [95]
Transfected with Erβ	E2 (10 nM) for 24 h	Inhibited cell proliferation Arrested cell cycle at G_1_ phase	↓ c-MYC expression ↑ p21^(Waf1/Cip1)^ expression ↑ p27^Kip1^ expression *Via suppressing the F-box protein p45(Skp2) expression*	Hartman et al., 2009 [97]
Transfected with Erβ	E2 (10 nM) for 24 h	Regulated cell cycle & anti-inflammatory response	↓ MYC, MYB, PROX1 oncogenes expression ↓ IL-6 cytokine expression	Edvardsson et al., 2011 [105]
Transfected with Erβ	-	# No effect on cells proliferation, apoptosis & cell cycle. Increased/enhanced invasion Inhibited cell migration	-	Tu et al., 2012 [106]
-	Raloxifene (10 µM) for 48 h	# No effect on cells proliferation Induced apoptosis (6%)	-	
Transfected with Erβ	Raloxifene (10 µM) for 48 h	Synergistically inhibited cell proliferation Synergistically induced apoptosis Synergistically decreased invasion Synergistically decreased cell migration	-	
HCT116 cells transfected with ERβ In Vivo (mice) Xenograft model. Injected with HCT116 cells	Raloxifene (0.5 mg/mouse every 3 days) For 40 days therapy	Synergistically inhibited cells proliferation Synergistically induced apoptosis/necrosis	-	
Silenced/depleted of endogenous ERα	-	Inhibited cell proliferation Arrested cell cycle at the G_1-_S phase Inhibited colony formation	↓ CDK2 activity expression ↓ Hyperphosphorylated state of Rb ↑ p21^(Waf1/Cip1)^ expression ↑ p27^Kip1^ expression	Bernatchez et al., 2013 [104]
Silenced/depleted of endogenous ERα In Vivo (mice) Xenograft model. Injected with HCT116 CRC cells	-	After 32 days after inoculation: Reduced the tumorigenic capacity	-	
Transfected with ERRα	Simvastatin (ERRα inhibitor) (10 μM) alone for 48 h	Inhibited cell growth. Inhibited colony formation	↓ c-MYC oncogene expression ↓ Cyclin D1 expression	Zhou et al., 2018 [107]
	Simvastatin (ERRα inhibitor) (10 μM) + Trametinib (MEK inhibitor) (50 nM) for 48 h	Increased the sensitivity of HCT116 cells to trametinib		
Transfected with ERRα in In Vivo (mice) Xenograft model. Nude mice subcutaneously injected with HCT116 cells into female BALB/c nude mice, 4–6 weeks old.	Simvastatin (ERRα inhibitor) treated daily orally with 1.5 mg/kg + Trametinib (MEK inhibitor) Treated daily orally with 1.5 mg/kg	Synergistically reduced xenograft tumor volume and weight	↓ ERRα expression ↓ c-MYC expression ↓ Cyclin D1 expression	
-	ERB-041 (ERβ-selective agonist) (60 nM) for 24 48, or 72 h	Inhibited cells proliferation Reduced colony formation Reduced cell migration Induced apoptosis	↑ ERβ nuclear expression ↓ CYST_2_R nuclear expression ↓ Cyclin D1 nuclear expression ↓ MYC nuclear expression ↓ Active β-catenin expression ↑ Caspase-3 activity expression	Topi et al., 2020 [88]
-	ERB-041 (ERβ-selective agonist) into the perivitelline space of Zebrafish Xenograft model (in vivo) Injected with HT116 cells for 48 h	Reduced cells metastasis through increased the expression of ERβ	-	Topi et al., 2020 [88]

Symbols: ↑—increase; ↓—decrease; #—no effect; grey shading indicates in vivo experiment.

**Table 5:** Summary of studies that measured the effects of estrogen and/or its related receptors in SW480 cells ^(ERα^^−^^& ERβ+)^ in chronological order.

Treatment Strategy		Potential Actions	Suggested Mechanisms	References
Gene Insertion/Deletion	Exogenous Treatment			
Transfected with ERβ	E2 (10 nM) for 24 h	Inhibited cell proliferation Arrested cell cycle at G_1_ phase	↓ c-MYC expression ↑ p21^(Waf1/Cip1)^, p27^Kip1^ expression *Via suppressing the F-box protein p45(Skp2) expression*	Hartman et al., 2009 [97]
Transfected with ERβ In Vivo (mice) Xenograft model. Injected with SW480 cells	E2 (0.72 mg/pellet) for 14 days	Reduced 70% of tumor growth Arrested cell cycle at G_1_ phase	-	Hartman et al., 2009 [97]
Transfected with ERβ	E2 (10 nM) for 24 h	Regulated cell cycle Regulated the anti-inflammatory response	↓ MYC, MYB, PROX1 oncogenes expression ↓ IL-6 cytokine expression	Edvardsson et al., 2011 [105]
Transfected with ERβ	E2 (1–10 nM) for 24 h	Inhibited cells proliferation Induced apoptotic cell death Induced DNA damage Impaired cell migration	↓ MYC oncogenes expression ↓ miR-17–92 cluster ↓ miR-200a/b ↓ E-cadherin expression	Edvardsson et al., 2013 [108]
Transfected with ERβ	E2 (10 nM) for 24 h	# No effect	-	Nguyen-Vu et al., 2016 [109]
Transfected with ERβ	-	Inhibited cells proliferation Arrested cell cycle at G_0_ phase Increased cell adhesion Reduced cell invasion Impaired cell metastasis	↑ miR-205 ↓ PROX1 oncogene expression	
Transfected with ERRα	Simvastatin (ERRα inhibitor) (10 μM) Alone for 48 h	Inhibited cell growth. Inhibited colony formation	↓ c-MYC oncogene expression ↓ Cyclin D1 expression	Zhou et al., 2018 [107]
	Simvastatin (ERRα inhibitor) (10 μM) + Trametinib (MEK inhibitor) (50 nM) for 48 h	Increased the sensitivity of HCT116 cells to trametinib		
Transfected with ERRα In Vivo (mice) Xenograft model. Nude mice subcutaneously injected with HCT116 cells into female BALB/c nude mice, 4–6 weeks old.	Simvastatin (ERRα inhibitor) treated daily orally with 1.5 mg/kg + Trametinib (MEK inhibitor) Treated daily orally with 1.5 mg/kg	Synergistically reduced xenograft tumor volume and weight	↓ ERRα expression ↓ c-MYC expression ↓ Cyclin D1 expression	

Symbols: ↑—increase; ↓—decrease; #—no effect. Grey shading indicates in vivo experiment.

**Table 6:** Summary of studies that measured the effects of estrogen and/or its related receptors in SW620cells^(ERα+& ERβ+)^ in chronological order.

Treatment Strategy		Potential Actions	Suggested Mechanisms	References
Gene Insertion/Deletion	Exogenous Treatment			
Transfected with ERβ	E2 (1–10 nM) for 24 h	Inhibited cells proliferation	# No effect on MYC and downstream miRNAs	Edvardsson et al., 2013 [108]
Transfected with ERβ	E2 (10 nM) for 24 h	# No effect	-	Nguyen-Vu et al., 2016 [109]
Transfected with ERβ	-	Inhibited cells proliferation Arrested cell cycle at G_0_ phase Increased cell adhesion	↑ miR-205 expression ↓ PROX1 oncogene expression	

Symbols: ↑—increase; ↓—decrease; #—no effect.

**Table 7:** Summary of studies that measured the effects of estrogen and/or its related receptors in LoVo cells^(ERα^^−^^/+ & ERβ+)^ in chronological order.

Treatment Strategy		Potential Actions	Suggested Mechanisms	References
Gene Insertion/Deletion	Exogenous Treatment			
-	E2 (10^−8^–10^−6^ M) for 48 h	Inhibited cell proliferation	-	Lointier et al., 1992 [110]
-	E2 (10 nM–1 µM) for 48 h	Inhibited cell proliferation	-	Fiorelli et al., 1999 [90]
-	E2 (1 to 500 nM) for 96 h	# No effect on cell proliferation	-	Arai et al., 2000 [94]
Transfected with ERβ	E2 (10^–8^ M) for 12–16 h.	Inhibited cell proliferation Arrested cell cycle at G_1_ and S phases. Induced apoptosis Induced DNA fragmentation	↑ p21^(Waf1/Cip1)^ expression ↑ p27^Kip1^ expression ↓ β-catenin, cyclin D1/E, and Rb expression ↑ p53 expression # TNF-*α* expression ↑ Caspase-8 and -9 activity	Hsu et al., 2006a [111]
Transfected with ERᶐ	E2 (10^–8^ M) for 12 h.	Inhibited cell proliferation Arrested cell cycle at G_1_ and S phases. Induced apoptosis Induced DNA fragmentation	↑ p21^(Waf1/Cip1)^ expression ↑ p27^Kip1^ expression ↓ β-catenin, cyclin D1/E, and Rb expression ↑ TNF-*α* expression, ↓ p53 expression ↑ Caspase-8, -9, and -3 activity	Hsu et al., 2006b [112]
-	E2 (10^−8^ M) for 30 min, followed by PGE2 (10^−6^ M) treatment for additional 3 or 24 h	Impaired cell migration	↓ COX-2 expression ↓ AKT and ERK1/2 activation	Lai et al., 2010 [113]
-	E2 (10^−8^ M) for 30 min or 1 h, followed by PGE2 (10^−6^ M) treatment for additional 24 or 48 h	Impaired cell migration	↓ uPA and MMP-9 expression ↓ JNK1/2 activation	Hsu et al., 2011 [114]
Transfected with ERs (α, β)	E2 (10^−8^ M & 10^−9^ M) for 24–48 h	Inhibited cell proliferation Impaired cell migration	↓ Cyclin A/D1/E expression Via p38/MAPK activation ↓ uPA and tPA expression ↓ MMP-2 and MMP-9 expression	Hsu et al., 2012 [115]
-	E2 and/or ER agonists (10 ^−9^ M & 10^−8^ M) for 24–48 h	Inhibited cell proliferation Impaired cell migration	↑ p53 expression ↑ p21^(Waf1/Cip1)^ expression ↑ p27^Kip1^ expression ↓ Cyclin D1 expression ↓ uPA and tPA expression ↓ MMP-2 and MMP-9 expression	Hsu et al., 2014 [116]

Symbols: ↑—increase; ↓—decrease; #—no effect.

**Table 8:** Summary of studies that measured the effects of estrogen and/or its related receptors in HCT8 cells^(ERα^^−^^& ERβ+)^ in chronological order.

Treatment Strategy		Potential Actions	Suggested Mechanisms	References
Gene Insertion/Deletion	Exogenous Treatment			
-	E2 (10 nM) for 48 h	# No effect on cell proliferation	-	Fiorelli et al., 1999 [90]
Transfected with ERβ	E2 (10 nM) for 24 h	Inhibited cell proliferation Arrested cell cycle at G_1_-S phases	↑ p21^(Waf1/Cip1)^ expression ↓ Cyclin E expression	Martineti et al., 2005 [117]

Symbols: ↑—increase; ↓—decrease; #—no effect.

**Table 9:** Summary of studies that measured the effects of estrogen and/or its related receptors in COLO205 cells^(ERα^^−^^& ERβ+)^ in chronological order.

Treatment Strategy		Potential Actions	Suggested Mechanisms	References
Gene Insertion/Deletion	Exogenous Treatment			
-	E2 (10^−12^, 10^−10^ 10^−7^ M) for 48 h	Induced apoptosis Induced DNA fragmentation	-	Qui et al., 2002 [73]
-	E2 (10^−11^, 10^−12^ M) for 24–48 h	Inhibited cells proliferation Induced apoptosis	↓ PKB & AKT expression ↓ MYC oncogene expression ↓ K-RAS expression ↓ Rb expression ↑ GADD153 expression ↓ VEGF & ERBB-3 expression. others	Qui et al., 2004 [118]
-	E2 (10^−11^ to 10^−7^ M) for 24 h	Inhibited cells proliferation Induced late apoptosis Induced DNA fragmentation	↓ c-MYB oncogene expression ↓ BCL-2 anti-apoptotic protein expression	Wilkins et al., 2010 [119]

Symbols: ↑—increase; ↓—decrease.

**Table 10:** Summary of studies that measured the effects of estrogen and/or its related receptors in HT29 cells^(ERα^^−^^/+ & ERβ+)^ in chronological order.

Treatment Strategy		Potential Actions	Suggested Mechanisms	References
Gene Insertion/Deletion	Exogenous Treatment			
-	E2 (1 to 500 nM) for 96 h	# No effect on cell proliferation	-	Arai et al., 2000 [94]
-	E2 (20 nM) for 48 h	# No effect on cell proliferation	-	Tanaka et al., 2008 [95]
Transfected with ERβ	E2 (10 nM) for 24 h	# No effect on cell proliferation Arrested cell cycle at G_1_ phase	↓ c-MYC oncogene expression ↑ p21^(Waf1/Cip1)^ & p27^Kip1^ expression *Via suppressing the F-box protein p45(Skp2) expression*	Hartman et al., 2009 [97]
Transfected with ERα46	E2 (10^−8^ mol/L) for 48 h	Inhibited cell proliferation Arrested cell cycle at G_0_/G_1_ phase. Induced Apoptosis	-	Jiang et al., 2008 [77]
Transfected with ERβ	E2 (10 nM) for 24 h	Regulated cell cycle & anti-inflammatory response	↓ MYC oncogene expression ↓ MYB oncogene expression ↓ PROX1 oncogene expression ↓ IL-6 cytokine expression	Edvardsson et al., 2011 [105]
Silenced/depleted of endogenous ERRα	-	Inhibited cell proliferation Arrested cell cycle at G_1_ and S phases	↓ CDK2 expression ↓ Hyperphosphorylated state of Rb ↑ p21^(Waf1/Cip1)^ & p27^Kip1^ expression	Bernatchez et al., 2013 [104]
Transfected with ERβ	-	Inhibited cells proliferation Arrested cell cycle at G_0_ phase Increased cell adhesion Reduced cell invasion Impaired cell metastasis	↑ miR-205 expression ↓ PROX1 oncogene expression	Nguyen-Vu et al., 2016 [109]
-	ERB-041 into the perivitelline space of Zebrafish Xenograft model (in vivo) Injected with HT29 cells for 48 h	Reduced cells metastasis through increased the expression of ERβ	-	Topi et al., 2020 [88]

Symbols: ↑—increase; ↓—decrease; #—no effect. Grey shading indicates in vivo experiment.

**Table 12:** Summary of studies that measured the effects of the combination treatment effect of estrogens and progesterone in CRC in vitro cells/in vivo models in chronological order.

CRC Cells/Models	Treatment Strategy		Potential Actions	Suggested Mechanisms	References
	Gene Insertion/Deletion	Exogenous Treatment			
In Vitro: HCT116, HT29	Transfected with PR	E2 + MPA (20 nM each) for 48 h	Inhibited cell proliferation	-	Tanaka et al., 2008 [95]
		E2 + P4 (20 nM each) for 48 h	# No effect on proliferation		
In Vivo: Sprague–Dawley female rats with CRC	-	E2 + P4 (60 μg/kg and 10 mg/kg) Injected the rats twice a week, observed at 84 h after the last injection	Inhibited cell proliferation Induced apoptosis	↑ Caspase-8 expression ↑ Caspase-3 expression ↑ PARP expression	Sasso et al., 2019 [125]

Symbols: ↑—increase; #—no effect. Grey shading indicates in vivo experiment.

## Data Availability

Not applicable.

## Abbreviations

**CRC**—colorectal cancer; **E2:**17b—estradiol; **ERβ**—estrogen receptor beta; **ER****α**—estrogen receptor alpha; **PR**—progesterone receptor; **CIMP**—CpG island methylator phenotype; **CIN**—chromosomal instability; **MSI**—microsatellite instability; **APC**—adenomatous polyposis coli; **CTNNB1**—catenin Beta 1, a protein coding gene; **KRAS**—Kirsten Ras protooncogene; **BRAF**—a human gene that encodes a protein called B-Raf; **SMAD4**—small mothers against decapentaplegic homolog 4; **TGFBR2**—transforming growth factor-beta receptor 2; **TP53**—tumor protein 53; **PIK3CA**—phosphatidylinositol-4,5-bisphosphate 3-kinase catalytic subunit-alpha; **Wnt**—Wingless/Int; **β-catenin**—beta-catenin; **EGFR**—epidermal growth factor receptor; **VEGF**—vascular endothelial growth factor; **RAS**—rat sarcoma virus; **RAF**—rapidly accelerated fibrosarcoma; **MAPK**—mitogen-activated protein kinase; **TGF-β**—transforming growth factor-beta; **PI3K**—phosphatidylinositol-4,5-bisphosphate 3-kinase; **AKT**—alpha serine/threonine protein kinase (protein kinase B); **BMI**—body mass index; **SHBG**—sex-hormone-binding globulin; **E**—estrogen; **E1**—estrone; **E3**—estriol; **P4**—progesterone; **ERs**—estrogen receptors; **PRs**—progesterone receptors; **PRA**—progesterone receptors N-terminal-truncated A; **PRB**—progesterone receptor full-length B; **HRT**—hormone replacement therapy; **OC**—oral contraceptive; **MPA**—medroxyprogesterone acetate; **ERR****α**—estrogen-receptor-related receptor alpha; **Caspase-3**—cysteine aspartic acid specific protease 3; **PARP**—poly (ADP-ribose) polymerase; **p38/MAPK**—p38 mitogen-activated protein kinase (a member of the mitogen-activated protein kinase family); **CDK4**—cyclin-dependent kinase 4; **p21^(Waf1/Cip1)^**—cyclin-dependent kinase inhibitor; **PCNA**—proliferating cell nuclear antigen; **ERK**—extracellular signal regulated kinase; **AKT**—protein kinase B; **NFκB**—nuclear factor κB; **CDK2**—cyclin-dependent kinase 2; **Rb**—retinoblastoma; **p21^(Waf1/Cip1)^ and p27^Kip1^**—cyclin-dependent kinase inhibitors; **NGB**—neuroglobin; **MYC**—myelocytomatosis oncogene; **MYB**—myeloblastosis oncogene; **PROX1**—prospero homebox 1; **IL-6**—interleukin- 6; **ERB-041**—ERβ-selective agonist; **CysT_2_R**—cysteinyl leukotriene receptor 2; **15-PGDH**—15-hydroxyprostandlin dehydrogenase; **miRNA**—microRNA; **Caspase-8 and -9**—cysteine aspartic acid specific protease 8 and 9; **P53**—tumor protein 53; **TNF-α**—tumor necrosis factor-alpha; **uPA**—urokinase plasminogen activator; **tPA**—tissue plasminogen activator; **MMPs**—matrix metallopeptidases; **JNK**—c-Jun N-terminal kinase; **COX-2**—cyclooxygenases; **GADD153**—growth arrest and DNA-damage-inducible protein 153; **PKB**—protein kinase B; **BCL2**—B-cell lymphoma 2; **ERBB-3**—epidermal growth factor receptor 3; **miR-205**—microRNA-*205*; **GADD45α**—growth arrest and DNA-damage-inducible protein α; **BAX**—BCL2-associated X apoptosis regulator.
